# The Olfactory System as Marker of Neurodegeneration in Aging, Neurological and Neuropsychiatric Disorders

**DOI:** 10.3390/ijerph18136976

**Published:** 2021-06-29

**Authors:** Naina Bhatia-Dey, Thomas Heinbockel

**Affiliations:** Department of Anatomy, Howard University College of Medicine, Washington, DC 20059, USA; naina.bhatiadey@howard.edu

**Keywords:** synaptic transmission, olfactory bulb, limbic system, GABA-A, GABA-B and dopamine receptors, mitral and tufted cell layers, neurodegenerative pathology, periglomerular cell

## Abstract

Research studies that focus on understanding the onset of neurodegenerative pathology and therapeutic interventions to inhibit its causative factors, have shown a crucial role of olfactory bulb neurons as they transmit and propagate nerve impulses to higher cortical and limbic structures. In rodent models, removal of the olfactory bulb results in pathology of the frontal cortex that shows striking similarity with frontal cortex features of patients diagnosed with neurodegenerative disorders. Widely different approaches involving behavioral symptom analysis, histopathological and molecular alterations, genetic and environmental influences, along with age-related alterations in cellular pathways, indicate a strong correlation of olfactory dysfunction and neurodegeneration. Indeed, declining olfactory acuity and olfactory deficits emerge either as the very first symptoms or as prodromal symptoms of progressing neurodegeneration of classical conditions. Olfactory dysfunction has been associated with most neurodegenerative, neuropsychiatric, and communication disorders. Evidence revealing the dual molecular function of the olfactory receptor neurons at dendritic and axonal ends indicates the significance of olfactory processing pathways that come under environmental pressure right from the onset. Here, we review findings that olfactory bulb neuronal processing serves as a marker of neuropsychiatric and neurodegenerative disorders.

## 1. Introduction

The mammalian olfactory system, the prime sensory modality for odor detection, is widely involved in detecting and processing olfactory signals. Processing of these sensory signals obtained from diverse chemical stimuli plays a pivotal role in physiological homeostasis. In addition, the mammalian sense of olfaction is a significant component of emotional homeostasis due to its critical role in reproductive and neuroendocrine regulation. In mammals, olfactory sensing begins in nasal olfactory structures and proceeds to the first central relay for olfactory processing, namely, the olfactory bulb [1,2,3]. In rodents, the olfactory bulb is a large structure occupying a quarter of the cranial cavity [4], whereas, in humans, it presents itself as a small extension of the brain. This cortical brain structure is a master sensory processor in mammals as it possesses all the features of the mammalian brain in a small space [5] with the exclusive allocation of its cell layers for processing odorant information [1,2,3]. Thus, the olfactory bulb is the first sensory structure for the direct contact of sensory stimuli and environmental pathogens that may impart varied impacts on neuronal types of its specific cell layers [6]. Experimental evidence suggests a concomitant role of olfactory bulb neurons in the onset of decline in olfactory acuity as well as the initiation of neurodegenerative pathology [7]. Conversion of olfactory stimuli into electrical signals occurs primarily through multiple pathways of olfactory bulb cell layers prior to conveying the signal to higher-order brain structures for further processing [8]. Olfactory receptor neurons (ORNs) undergo specific adaptations at the anatomical, physiological and functional levels based on chronic exposure to stimuli [9,10]. Odor-evoking environmental stimuli alter the activation of the autonomic nervous system through a decline of olfactory processing, stress perception, and subsequent physiological impact [11,12,13]. In humans, the sense of olfaction has a significant influence on day-to-day behavior and quality of life [14]. Impaired olfaction, together with a decline in olfactory acuity, has been suggested as a preliminary indicator of classical neurodegenerative disorders such as Alzheimer’s disease (AD) and Parkinson’s disease (PD). It is now recognized as prodromal symptom neuropathology associated with both conditions [7,15,16]. We review and analyze the literature revealing insights into the fine-tuning of sensory processing in the olfactory system that points to the existence of novel yet unidentified pathways for neuromodulation in olfactory and limbic structures with a potential contribution to the onset of neurodegenerative pathology.

## 2. The Olfactory Pathway

**Figure 1:**
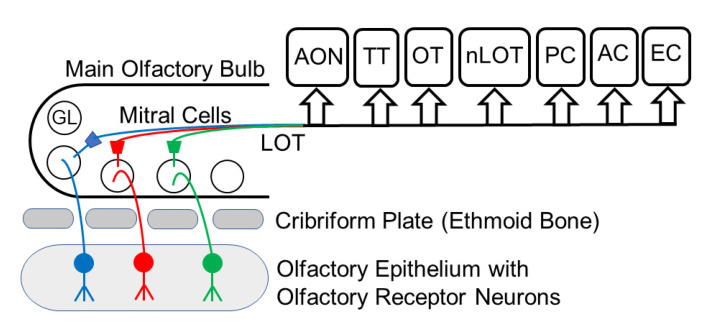
Schematic representation of the olfactory pathway. The olfactory pathway starts with the olfactory epithelium in the nose. Olfactory receptor neurons are bipolar nerve cells that send an axon each through the cribriform plate of the ethmoid bone to the ipsilateral main olfactory bulb. Olfactory receptor neurons synapse on mitral cells and other cells in olfactory glomeruli. Mitral cells send their axons through the lateral olfactory tract to higher-order olfactory centers. The main olfactory bulb houses additional neuron types that are not shown for clarity. The size of the boxes in the olfactory cortex does not represent the actual space occupied by a particular brain region. AC, amygdaloid complex; AON, anterior olfactory nucleus; EC, entorhinal cortex; GL, glomerulus; LOT; lateral olfactory tract; nLOT, nucleus of lateral olfactory tract; OT, olfactory tubercle; PC, piriform cortex; TT, tenia tecta.

Genetic, physiological, and environmental factors specifically modulate odor perception and processing in both olfactory and limbic systems. Subsequent activation of the autonomic nervous system due to aberrant olfactory processing and perception of stress, as well as their overall impact on diverse physiological pathways through the entire lifespan, are among several causative factors contributing to the onset of neurodegenerative pathology [11,12,13]. Odor perception has been known to modulate cognition as it can evoke strong emotions and recall strong memories [17,18,19,20,21]. Odorant information traverses through the olfactory pathway that begins in the nasal cavity lined with an olfactory epithelium with a large population of ORNs that house specific odorant receptor proteins (Figure 1). ORNs respond to inhaled odorant molecules that activate receptor proteins localized in neuronal cilia. During development, each immature ORN shows low expression levels of multiple olfactory receptor genes [22,23]. This transient expression contrasts with mature ORNs that firmly follow the rule of one neuron—one expressed olfactory receptor gene [22,23]. ORNs expressing the same receptor protein send their axons to the same glomeruli in the main olfactory bulb to generate synaptic contacts with dendrites of interneurons and output neurons of mitral and tufted cells of the olfactory bulb. In comparison to the large number of ORNs, relatively few output neurons innervate each glomerulus in the olfactory bulb; this is an important checkpoint of olfactory processing. In turn, these output neurons send their axons to higher-order brain centers in cortical and limbic structures. It is primarily cortical regions that receive olfactory signals from the olfactory bulb for further processing [18,19]. The precision of contact by ORN axons to specific olfactory bulb glomeruli and its further representation in the brain are the key factors distinguishing odorant specific signal processing as a collaborative function of olfactory and limbic systems, their relationships, together with long-term and short-term impact [1,19]. Specifically, functional receptor molecules of ORNs respond to odorants at the dendrite end and act as guidance molecules at the axonal end, regulating olfactory processing [20]. The axons of ORNs aggregate to form the olfactory nerve and olfactory nerve layer of the olfactory bulb. At the entry-level of inhaled stimuli, the pseudostratified olfactory neuroepithelium is comprised of ORNs, supporting microvillar cells as well as essential basal stem cells. Basal stem cells replenish older short-lived ORNs in the neuroepithelium [24] with younger ORNs [25]. Axons of these epithelial ORNs travel through the cribriform plate and project into the glomerular layer of the olfactory bulb, where they synapse on mitral and tufted cells and juxtaglomerular cells. Mitral and tufted cells send their axons into olfactory cortical and limbic areas (Figure 1). Olfactory information reaches neocortical areas that are involved in higher-order sensory processing. The crucial role of the olfactory system is evident from studies of the onset of neurodegeneration that detect misfolded and/or unfolded proteins in the olfactory neuroepithelium that accompany early and prodromal symptoms of impaired olfaction prior to the appearance of neurodegenerative pathology [25]. As an example of how aging and neurodegeneration can be associated with somatic mutations in neurons, a study by Lodata et al. in hippocampal and prefrontal neurons shows that mutations in individual neurons increased with age and were more abundant in neurodegenerative diseases [26]. This study opens the door to explore how molecular signatures may be important in other human age-associated conditions and possibly other systems such as the olfactory system. Experimental evidence demonstrates that growth factor IGF1 (insulin growth factor1) induces glomerulus-specific long-term potentiation (LTP) at GABAergic (GABA, gamma aminobutyric acid) granule cell to mitral cell synapses in olfactory bulb cell layers as a component of odor-dependent social transmission of food preference or STFP (social transmission of food preference) [27]. While olfactory bulb-specific deletion of IGF1 receptors prevents LTP and impairs odor-related memory formation after STFP, the addition of IGF1 to olfactory bulb slices demonstrated GABAergic LTP [27]. These findings suggest that synaptic strength could play a critical role in the processing of odor-evoked sensory information. In the following sections, we review various factors affecting odor processing and transmission by neurons in the olfactory epithelium and olfactory bulb to the limbic system and cortical areas and structures involved in higher-order odor processing such as the anterior olfactory nucleus and piriform cortex. Our analysis is aimed at understanding the functional influence of suboptimal and/or aberrant odor processing that may indicate a concurrent decline in olfactory acuity and overall olfactory dysfunction together with the onset of physiological and pathological features related to neurodegeneration.

## 3. Aging and Olfaction

Declining olfactory acuity and olfactory dysfunction ranging from subtle to severe have now become a well-established feature of the normal aging process [28,29,30,31,32]. These are detectable in an age-dependent manner: in 50% of tested subjects ranging from 65 to 80 years in age, while increasing to 75% of those who are above 80 years [8,28,29,30,31,32,33,34,35]. Age-associated ossification and closure of foramina of the cribriform plate [34,36], as well as quantitative reduction of the olfactory epithelium and its replacement by respiratory epithelium [37], are anatomical and histological factors leading to olfactory decline and dysfunction. Additional contributing factors, include age-associated thinning of the olfactory neuroepithelium accompanied by marked alteration in cellular patterns and regional distribution of nuclei of olfactory receptors and sustentacular cells [6,34], as well as loss of olfactory receptor cells and reduction of mucosal metabolizing enzymes [35]. Moreover, a decline in the volume of the olfactory bulb with increasing chronological age of both genders occurs with a parallel loss of olfactory acuity and onset of olfactory dysfunction [38,39,40]. Reduced proliferation, as well as a reduction in the number of ORNs, causes a decline in expression of extracellular matrix genes such as *Insulin growth factor1* (*Igf1*) and leads to increased senescence-associated secretory phenotype (SASP) that contributes to olfactory impairment during aging [41]. Experimental evidence using magnetic resonance imaging (MRI) for healthy normal aging individuals also indicates an association of olfactory loss with a reduction in volume and morphological impairment of olfactory structures of the limbic system, such as the amygdala, entorhinal, and perirhinal cortex, as well as certain cerebral regions that do not participate directly in olfactory processing [42]. In addition, there are notable age-related decreases in cerebral cortical thickness, myelination, and synapse number accompanied by ex vacuo enlargement in ventricular volume [43]. Using single nucleus ribonucleic acid (RNA) sequencing, a population of AD pathology associated astrocytes is detectable in the aging human brain and in the brains of a mouse model of AD as well as in aging mice [32,44]. When sensory neurons, isolated from older and younger subjects, are exposed to odorant mixtures, loss of sensitivity and specificity is detectable in neurons from older subjects [45]. Further experimental evidence using pleasant odorants in older individuals to test for olfactory responses reveals their “less pleasant” rating and reduced beta-event-related synchronization. Together these findings suggest a decline in olfactory processing [11]. A study utilizing a high frequency of naturally occurring olfactory receptor knockouts in the human genome shows that loss-of-function of single olfactory receptors alters odor perception, thereby affecting odor identification [46]. The odor identification (OI) scale is now considered as an indicator of the mortality rate of the aging population. A lower OI in the septuagenarian community (70–79 years of age) correlates with an increased risk of dementia [47], and a reduced OI has been linked to advanced physiological brain aging and onset of neurodegenerative pathology [48]. However, one study using hyposmic and anosmic subjects of both genders in their very early seventies, did not detect any association of olfaction with asymptomatic amyloidosis and cognition [49].

## 4. Neuropsychiatric Disorders

According to recently developed Health Research domain criteria [RDoC] for neuropsychiatric disorders by the National Institute of Mental Health, olfactory function has been included in the cognitive domain of the brain function assessment [50]. Apart from healthy aging individuals and patients diagnosed with neurodegenerative disorders, impaired olfaction has been reported in mild cognitive impairment (MCI), frontotemporal dementia (FTD), and a range of depressive disorders [51,52,53]. Multiple studies indicate that olfactory deficits and low OI scores are characteristic features of mild to severe major depressive disorders [53,54]. Loss of normal olfaction has emerged as a distinct marker of depression in a comparative analysis of patients diagnosed with depression and age-matched control individuals [55]. A correlation has emerged as patients diagnosed with depression display reduced olfactory performance, whereas patients with olfactory dysfunction display worsening depression symptoms that are comparatively more acute in anosmic subjects than in hyposmic subjects [56]. Patients diagnosed with post-traumatic stress disorder (PTSD) and major depressive disorder (MDD) display olfactory dysfunction and decline. MDD patients show a negative impact on both primary and secondary olfactory processing [56,57,58]. MDD patients show reduced activation in the thalamus, insula, and left middle orbitofrontal region. These are considered secondary olfactory structures and, therefore, odor processing here is defined as secondary olfactory processing. The piriform cortex is a primary region of olfactory processing and/or its fine-tuning, and, therefore, olfactory processing in the piriform cortex is considered primary olfactory processing. In patients with a diagnosis of a behavioral FTD variant, initially OI and odor discrimination remained the same as control cases, but their significant difference in an odor association test has been attributed to impaired olfactory processing [59]. A later study conducted to define olfactory dysfunction with precision used comparative analysis in patients with FTD, depression, schizophrenia, and bipolar disorder. It revealed that FTD patients were severely impaired in OI but not in odor discrimination [60]. Based on environmental exposure, encoded olfactory stimuli activate emotional memory [61]. Due to the anatomical link of the olfactory system with brain circuits participating in memory and cognition, such stimuli further contribute to functional alteration in patients with depression [62,63,64].

Patients with autism spectrum disorders (ASDs) and bipolar disorder also show a decline in olfactory function and acuity [65,66]. ASDs are clinically heterogeneous polygenic disorders with complex genetic etiology [67,68]. The majority of neuropsychiatric disorders have a prominent genetic component that is susceptible to de novo variation leading to an enhanced possibility of functional deficits through altered developmental processes during differentiation and formation of the nervous system [69]. All 102 identified genes with an established contribution to increased ASD risk show de novo variations either in individuals with neurodevelopmental delay or with ASD. Most genes have a regulatory role in gene expression and neuronal communication that affect neurodevelopmental and neurophysiological alterations [70]. Moreover, they are expressed and enriched early in excitatory and inhibitory neuronal lineages and affect synapses [70]. In ASD mouse models, experimental analysis reveals weaker and fewer synapses between olfactory sensory nerve terminals and olfactory bulb tufted cells as well as inhibitory periglomerular cells in the olfactory bulb [71]. Whole-genome-sequencing analysis of an autistic proband at age 10 along with a 12-year old healthy sibling revealed the presence of a non-sense mutation of *ANOS1* gene [68]. The ANOS1 protein has a role in axonal guidance and migration of olfactory neurons [72]. Duplication of *GABA* receptor genes and deletion of *TOP3B* (*DNA Topoisomerase III Beta* is a protein-coding gene), a topoisomerase involved in the relaxation of supercoiled DNA, contribute to autism susceptibility. They belong to gene families with a specific contribution to multiple neurodevelopmental disorders, including schizophrenia [73]. While duplicated *GABA* receptor genes might contribute to irregularity in synaptic strength and/or neurotransmitter function, *TOP3B* deletion is likely to alter neuromodulation through perturbed gene regulation and/or epigenetic misregulation.

## 5. Neurodegenerative Diseases

Any marginal change in olfactory perception reflects subtle olfactory dysfunction that frequently precedes a number of neurodegenerative disorders and is presumed to be due to loss of synaptic function [74,75,76]. Odors as environmental stimuli have a significant influence on autonomic nervous system activation induced by a combination of factors that affect olfactory processing, perception of stress, and downstream molecular pathways associated with such activation [11,12,13]. Acceleration of environmental impact leads to cellular senescence and related physiological alterations in diverse cellular pathways, which are part of the evolving homeostasis as a function of chronological age [77,78]. Added years of age contribute to gradual changes in the physiological milieu based on genetic and environmental factors and increase the risk for neurodegeneration [78]. Cellular senescence, defined as causal nexus of aging [77,79], contributes to the progression of many health-related conditions, including AD and other diseases of neurodegenerative pathology [80,81]. As such, brain tissue continues to accumulate somatic mutation in both proliferative and non-proliferative cell types through lifetime [82]. Together with these, slowly altering genetic and epigenetic factors are in place that are impacted by demographics, lifestyle, and the addition of chronological years. One example of a genetic factor is the presence of an unusually high genetic diversity of the human olfactory receptor gene family that leads to a highly personalized inventory of functional olfactory receptors in the human population [83]. While cellular senescence marks the end of the proliferative cellular phase, age-associated accumulation of senescent cells leads to SASP that may spread senescence to nearby and distant cells [79]. Using genome-wide single nucleotide variant analysis, an age-dependent accumulation of somatic mutations at the single neuron level is detectable in normal aged individuals and those diagnosed with early onset of neurodegenerative disorders [26]. A variable range of olfactory dysfunction is clearly evident in AD, PD, progressive supranuclear palsy (PSP), frontotemporal degeneration (FTLD-YDP43), and mild cognitive impairment (MCI) [19,84,85,86,87,88,89]. In a recent comparative meta-analysis of over 1100 patients, the olfactory performance of PD patients was worse than patients with PSP and other neurological disorders [90]. At the cellular level, the relationship of olfactory function with neurodegeneration is underscored by the presence of four characteristic neurodegeneration-associated proteins: α-synuclein, transactive response DNA-binding protein 43 (TDP-43), hyperphosphorylated tau and β-amyloid proteins in olfactory neurons and mucosa [25]. The clinical diagnosis of AD relies on cognitive deficits and, particularly, on anterograde amnesia [91,92,93]. AD patients have impaired olfaction accompanied by an accumulation of hyperphosphorylated tau and β-amyloid proteins in the olfactory system [94]. The accumulation of neuritic plaques of hyperphosphorylated tau and β-amyloid proteins towards AD neuropathology follows a six-stage deposition pattern that begins in locus coeruleus [first stage], olfactory bulb, and entorhinal cortex (second and third stages, respectively), extending subsequently to the temporal cortex and other regions that are defined as higher stages [fourth, fifth, and sixth stages, respectively] of AD neuropathology [93].

While no change in olfactory bulb cell numbers in the mitral cell layer in AD patients has been found, there is a significant reduction in olfactory bulb volume and an increase in periglomerular dopaminergic cells in the anterior olfactory nucleus, compared to normal aging controls [95,96]. The prime feature of the neurodegenerative pathology of PD is a reduction of dopaminergic neurons, resulting in a gradual but significant loss of dopamine that finally results in many clinical motor symptoms such as bradykinesia, rigidity, tremor, instability of posture, and decline of cognitive function [97]. Olfactory dysfunction is detectable in 90% of preclinical PD cases, preceding the onset of motor symptoms by decades [98]. Hyposmia is correlated with at least a 10% increased risk of developing PD [99]. In PD patients, olfactory dysfunction also shows a correlation with degenerative features of olfactory-specific regions of the right amygdala and piriform cortex [100] and atrophy of the orbitofrontal cortex [101]. At the histopathological level, PD patients show aggregates of Lewy bodies and Lewy neurites, comprised of α-synuclein that first appear in cholinergic and monoaminergic brainstem neurons and in neurons in the olfactory system, including the olfactory bulb. Eventually, with disease progression, they are detected in limbic and isocortical regions as well as other organ systems, which indicates the complexity of pathways contributing to the diverse PD symptoms, specifically dementia and severe hyposmia [102,103,104,105]. In parallel with progressive Lewy body pathology, there is a profound cell loss in the olfactory bulb and olfactory tract. This is specifically pronounced in the anterior olfactory nucleus [106] and is accompanied by an increase in dopaminergic cells that contribute to progressive hyposmia without overall loss in olfactory bulb volume [107], and that is in contrast to AD (see above).

In humans and all primates, the anterior olfactory nucleus comprises at least seven divisions along the olfactory system: bulbar, intrapeduncular, retrobulbar, anterior and posterior cortical portions, as well as lateral and medial components that show a preferential impact of proteinopathies [93,108,109,110]. Comparison of the spatial expression of neuronal markers in various neuronal populations of PD patients and age-matched controls with quantitative immunostaining indicates a high level of calcium-binding protein parvalbumin throughout neuronal cell bodies of the anterior olfactory nucleus, especially in its cortical division in PD patients [111]. Moreover, the retrobulbar division of PD patients shows an enhanced expression of two additional calcium-binding proteins, calbindin, and calretinin as well as parvalbumin, and the cortical posterior division indicates only enhanced calretinin expression [112]. Co-localization analysis of these marker proteins with α- synuclein and using non-calcium binding protein somatostatin as control revealed that α- synuclein localized with calcium-binding proteins and only partially with non-calcium binding somatostatin [111]. Together these findings indicate differential vulnerability of interneuronal population of the anterior olfactory nucleus towards α-synucleinopathy [111]. In AD, a decline in somatostatin expression in the anterior olfactory nucleus co-localizes with the accumulation of β-amyloid proteins [112]. Such quantitative and qualitative differences are also evident in non-neuronal cell populations [113]. A quantitative analysis of activated microglia and astrocytes in AD and PD patients along with age-matched healthy control individuals indicates an increase in activated microglia and astrocytes in both neurodegenerative conditions [60]. In PD patients, the anterior olfactory nucleus shows abundant α-synuclein aggregates in non-neuronal cells, including microglia and astrocytes [114]. Therefore, despite the differences in etiology and symptomatology of AD and PD patients at the behavioral and cellular levels, both neurodegenerative disorders have a long prodromal period that is marked by hyposmia in most patients and needs additional detailed analysis to delineate the role of different components of olfactory and limbic systems for a clearer understanding of neurodegeneration-related hyposmia [94]. Rapid eye movement sleep behavior disorder (RBD) is an additional well-established clinical marker of PD. It is marked by an increment in odor threshold and significant impairment in odor identification and discrimination [115]. However, RBD is a recently identified marker where additional physiological and molecular information is still very limited.

## 6. Olfactory Function, Memory, and Genes

In the preceding sections, we have reviewed and summarized research studies that indicate the pivotal contribution of various components of the olfactory system in odor processing and olfactory function. These two closely linked mechanisms maintain physiological and emotional homeostasis throughout the lifetime [12,13,42,55]. At the same time, their distinct decline is evident as a function of normal chronological age [28,29,33,34,35,116]. Age-associated anatomical, morphological and histological alterations affect odor processing in both olfactory and limbic systems [35,36,37,38,39,40]. Additionally, genetic and environmental factors regulate the process suggesting a genetic predisposition [46,117,118] and demographic influences [46]. A tissue-specific deletion of IGF1 receptor, specifically affecting the olfactory bulb, prevents glomerulus-specific LTP at the granule cell to mitral cell synapse of GABAergic neurons, indicating a direct link of odor processing and transmission with neural function [27]. Moreover, misregulation of gene expression due to the combined impact of environmental insults and genetic factors are powerful modifiers affecting odor processing and its transmission as regards neural function. Inherent allelic variations of brain-derived neurotrophic factor (BDNF) also affect and add to age-dependent olfactory decline [34,117]. One example of a genetic variation is the single nucleotide variations in *ANOS1* gene that produces ANOS1 protein, a participating molecule in migration of olfactory neurons and in axonal guidance [67,68,119]. A recent study indicated that ANOS1 protein activates vascular growth factor receptors and their related signaling pathways for angiogenesis in the olfactory bulb [120]. Therefore, the impact of single nucleotide variations in *ANOS1* gene on olfactory bulb mediated synaptic function could be due to aberrant function or inadequate activation of a crucial signaling pathway.

Out of 102 loci identified as contributing loci to enhanced risk factors for autism, most have regulatory functions affecting gene expression in early brain development or in neuronal communication by affecting neurophysiological processes through their impact on synaptic strength [70]. Duplication of *GABA* receptor genes increases autism susceptibility either through altered neurotransmitter function or irregularity of synaptic strength. Similar susceptibility, cognitive impairment, and behavioral dysfunctions also occur with deletion *TOP3B* locus that encodes for a topoisomerase involved in relaxing supercoiled DNA [73,121]. Indeed, *TOP3B* mutations have been proposed to play a role in shortened life span and onset of neurological disorders [122]. Thus, a small genetic variation causing a subtle modification of protein structure as well as one limiting the accessibility of regulatory regions such as promoter or enhancer contributes to neurodevelopmental disorders that have olfactory dysfunction as their first indicative symptom. A classic example in this regard is multiple sclerosis (MS), with a wide range (20–40%) of olfactory dysfunction [123]. A recent study, including a 10-year follow-up, indicates that olfactory impairment in MS patients is associated with disease severity and decreased survival time [124]. A crucial correlation has emerged with the early detection of olfactory dysfunction that precedes declining and deteriorating neural function in diverse neuropsychiatric and neurodegenerative disorders [74,75,76]. Indeed, studies exploring components of the olfactory system have provided valuable insight into neuropsychiatric behavior. Continuous exploration of mechanisms of olfaction is likely to unravel many unidentified aspects of neural function [125].

Age-based alterations in olfaction come under environmental pressure. Pollution in industrialized regions impairs olfaction when compared to people living in natural environments such as the rainforest [126]. Contact of the olfactory epithelium with air pollutants has a negative impact on the conveyance of odor information to neuronal synapses of olfactory bulb cells and its communication with the olfactory cortex and the limbic system. Moreover, pollutants distort the cellular morphology of the olfactory system and may cause inflammation as an outcome of the stress response [127]. Recent experimental evidence indicates that age-based alteration of gene expression may retain or modulate olfactory acuity [46]. A reduction in neurons with complex karyotypes is evident as a function of age, such that older individuals have fewer frontal cortex neurons with complex karyotypes in comparison to younger individuals [128]. At the same time, in a comparative quantitative analysis for the heritability of cognition and olfaction, the heritability of odor identification was lower, despite the contribution of the same genes in both traits [129]. Odor identification tests reliably define olfactory acuity and overall health status based on its reflection in olfactory function. A decline in olfactory acuity shows a correlation with dementia and progressing dysfunction [47,48]. At the symptomatic level, olfactory dysfunction is often accompanied by episodes of depression, anxiety, amnesia, as well as other cognitive and communication deficiencies [53,56,66]. These findings suggest that based on parallel behavioral anomalies, olfactory dysfunction together with distinct identifying clues may enable clinicians to distinguish between various neural disorders with overlapping symptoms. Continuation of olfactory deficits and/or their acceleration with altered behavior could suggest additional stepwise testing and systematic analysis to obtain pathophysiological clues for a relatively specific diagnosis. Accuracy of diagnosis will be helpful in early intervention to slow down disease progression and exploring combinatorial therapeutic approaches that may include talk therapy, lifestyle counseling, and novel drugs.

## 7. Conclusions

A review of the recent literature shows that olfactory dysfunctions are a powerful early marker of forthcoming neuropsychiatric, neurodegenerative, and communication disorders. The mammalian olfactory system has remained an interesting puzzle from an evolutionary standpoint due to its distinct functional differences in rodent models and humans [5,14]. This is clearly reflected in the variety and number of olfactory receptor proteins expressed by olfactory receptor neurons as there are approximately 1400 genes encoding olfactory receptor proteins in mice but around 400 in humans [83,130,131,132]. A hierarchical structure of the superfamily of olfactory receptor genes enables diverse adaptations to the chemical cues of the living environment with a relatively large number of gains and losses in the gene family as well as a considerable fraction of pseudogenes, about 300 in mice and 600 in humans [133,134]. In addition, there are also species-specific adaptations of the olfactory system to different ecological niches [135]. The olfactory system participates in the initial sensory processing right from the first exposure of ORNs in the nose to inhaled olfactory stimuli. As olfactory signals reach and traverse through the olfactory bulb cell layers, the central olfactory neurons serve as the first contact point in the brain for external stimuli/stressors. Mitral and tufted cells project their axons into olfactory areas that include the anterior olfactory nucleus, piriform cortex, amygdala, and entorhinal cortex before signals are transmitted to the orbitofrontal cortex and other associated regions for higher-order sensory processing. Altered physiological and molecular milieu, for example, through misfolded and unfolded proteins (7) and other factors, is plausible at multiple checkpoints of the olfactory system as a function of life experience and chronological age. Such alterations are yet to be discovered. However, they could potentially influence olfactory processing pathways from their onset. In addition, recent experimental evidence from mice indicates that together with odor detection, receptor molecules also define the target in the olfactory bulb, thereby modulating odor map and its specificity through the dual function: response to odor at the dendritic end and as olfactory bulb guidance molecules at the axonal end of the same neuron [20]. While regeneration in the peripheral olfactory system continues throughout the lifespan, age-related loss of neurodegenerative ability is evident in the mouse and human olfactory epithelium [136,137]. At the same time, experimental evidence shows a lack of olfactory memory deficits in AD mouse models that show extensive accumulation of β-amyloid proteins in olfactory memory regions of their brains [138]. These results are suggestive of a certain level of yet unidentified differences in the correlation of AD pathology with olfactory dysfunction in mice and humans. Similarly, in neurotoxin-induced mouse models of PD, there is a marginal interneuronal deposition of α-synuclein, despite a concomitant presence of olfactory dysfunction and neurodegeneration [139].

Moreover, recent evidence indicates a requirement of chemokine signaling for injury-induced neurogenesis in the mouse olfactory epithelium [140]. As such, the olfactory system serves as a gateway for prion-like propagation of odorants and pathogens as well as misfolded and unfolded proteins [7]. Each of its altered components and pathways are likely to interact with each other as well as with limbic system components, and together, they contribute to a decline in olfactory acuity, processing and function, neurodegenerative pathology as well as other behavioral and cognitive symptoms. A literature review examining the relationship of olfactory impairment with increased mortality risk of older populations indicates a clear association of olfactory impairment with advanced physiological brain aging as well as neurodegenerative diseases [48]. Based on the evidence of injury-induced neurogenesis in the olfactory epithelium, it is logical to presume that accumulated physiological deterioration, as an outcome of lifestyle, could negatively influence neurogenesis in the olfactory epithelium causing olfactory decline through multiple cell types. Emerging experimental evidence even indicates the participation of non-neuronal cells in olfactory processing and odor perception that precede neurodegenerative pathology [44,115]. Therefore, the overall evaluation of olfactory dysfunction in combination with additional symptoms while considering multiple environmental and genetic factors for added risk could provide avenues for early therapeutic intervention. There are multiple overlapping pathways of neuromodulation that could be explored for therapeutic intervention and drug designs [141]. A recent study using directly reprogrammed human neural precursor cell transplants in mice after deliberate stroke shows synaptogenesis upregulation and increased expression of synaptophysin in the ipsilesional hemisphere of the transplanted brain, thereby promoting functional recovery [142]. Such approaches could aid in developing specific therapeutic strategies for lowering the progression of neurodegenerative pathology, slowing of debilitating cognition/behavioral symptoms, and, finally, designing additional new therapeutic drugs with minimal side effects. Indeed, maintaining functional homeostasis of the olfactory system is a powerful approach to enhance the quality of life for individuals having a genetic disposition for neuropsychiatric and neurodegenerative conditions.

## Data Availability

Data sharing not applicable.
